# 

**Published:** 1910-12-17

**Authors:** 


### CONTENTS.

| | PAGE |
|---|---|
| **The King's Fund and Hospital Medical Schools** | 335 |
| **The New Parliament** | 336 |
| **Annotations—** An Advance Announcement—Medical Portraiture—The Beit Fellowships | 337 |
| **Medical Opinion and Movement** | 338 |
| **Hospital Clinics—** Orthopædics in General Practice. By J. Jackson Clarke, M.B. (London), F.R.C.S. | 341 |
| **Special Article—** Radiography | 345 |
| **Medicine—** The Ætiology of Hydrophobia | 346 |
| **Toxicology—** Carbon Monoxide Poisoning on a Motor Launch | 347 |
| **Practical Notes on Diagnosis and Treatment** | 348 |
| **News and Events of the Week** | 349 |
| **Appointments and Resignations** | 350 |
| **Editor's Letter-Box—** Quack Advertisements | 350 |
| **Obituary** | 350 |
| **"The Hospital" Medical Book Supplement—No. XXXVII** | 351 |
| **The Week's Work—** Lay Boards and Medical Committees—A West London Appointment—Dual Hospital Appointments—Hospital Medical Schools and the King's Fund—The Removal of Dangerously Sick Patients—The Christmas Appeal Number—This Week's Drug Market | 355 |
| **King Edward's Hospital Fund for London** | 357 |
| **Parliament and Publications** | 363 |
| **The Week in the Courts** | 363 |
| **The Hospital World** | 364 |
| **Institutional Notes and News** | 365 |
| **Gifts and Bequests** | 366 |
| **Jottings** | 366 |

